# A cohort study in family triads: impact of gut microbiota composition and early life exposures on intestinal resistome during the first two years of life

**DOI:** 10.1080/19490976.2024.2383746

**Published:** 2024-08-02

**Authors:** Roosa Jokela, Katariina MM Pärnänen, Alise J Ponsero, Leo Lahti, Kaija-Leena Kolho, Willem M de Vos, Anne Salonen

**Affiliations:** a Human Microbiome Research Program, Faculty of Medicine, University of Helsinki, Helsinki, Finland; b Department of Computing, University of Turku, Turku, Finland; c Children’s Hospital, University of Helsinki and Helsinki University Hospital (HUS), Helsinki, Finland; d Faculty of Medicine and Health Technology, Tampere University, Tampere, Finland; e Laboratory of Microbiology, Wageningen University, Wageningen, The Netherlands

**Keywords:** Infant resistome, early resistome, antibiotic resistance, infant microbiome

## Abstract

Antibiotic resistance genes (ARGs) are prevalent in the infant gut microbiota and make up the intestinal resistome, representing a community ARG reservoir. This study focuses on the dynamics and persistence of ARGs in the early gut microbiota, and the effect of early exposures therein. We leveraged 2,328 stool metagenomes from 475 children in the HELMi cohort and the available parental samples to study the diversity, dynamics, and intra-familial sharing of the resistome during the first two years of life. We found higher within-family similarity of the gut resistome composition and ARG load in infant-mother pairs, and between spouses, but not in father-infant pairs. Early gut microbiota composition and development correlated with the ARG load; *Bacteroides* correlated positively and *Bifidobacterium* negatively with the load, reflecting the typical resistance levels in these taxa. Caesarean delivered infants harbored lower ARG loads, partly reflecting the scarcity of *Bacteroides* compared to vaginally delivered. Exposure to intrapartum or post-natal antibiotics showed only modest associations with the ARG load and composition, mainly before 12 months. Our results indicate that the resistome is strongly driven by the normal development of the microbiota in early life, and suggest importance of longer evolution of ARGs over effects of recent antibiotic exposure.

## Introduction

Infant gut colonization starts at birth^1^ by bacteria from the mother and the environment. In addition to the live bacterial cells themselves, their genes are transmitted to the infant gut microbiota, contributing to the functional capacities of the developing intestinal community. A special interest on the transmission of antibiotic resistance genes (ARGs) has been on the rise for over a decade,^2,3^ as antibiotic resistance poses a risk on the vulnerable populations, like infants.^4^ Infant gut ARGs, or the resistome, originate from the same sources as infant gut microbiota in general, and distinct taxa tend to harbor ARGs at different rates.

Numerous factors have been identified to associate with the infant gut resistome by either increasing the general resistance gene load or by affecting individual genes. Delivery mode has been identified as a strong effector of the infant intestinal resistome, with Caesarean delivered infants both harboring larger proportions of ARGs than vaginally delivered infants^5^ and differing in resistome composition.^6^ The transmission of maternal bacteria is disrupted in Caesarean deliveries, where other bacteria from, *e.g*., skin or the hospital surfaces can be the first colonizers of the infant gut, and the transmission of maternal gut bacteria happens later.^7^ These early environmental colonizers often include pathogenic bacteria^8^ that are more likely to be ARG-carriers and can contribute to a higher ARG load. Additionally, formula feeding has been associated with higher resistance gene load than breastfeeding, although infants also receive ARGs from their mother through breastfeeding.^9^ A recent study suggests that certain bacterial genes can be transmitted even if the original host bacteria does not colonize the infant gut,^10^ suggesting that the early resistome can also vary independent from the microbiota composition.

The infant gut is first colonized with aerotolerant bacteria mainly from the *Enterobacteriaceae* family, but as the conditions in the intestines become anaerobic, genera such as *Bifidobacterium* and *Bacteroides* start to thrive.^11^ *Enterobacteriaceae* include many potential pathogens and have been identified as frequent ARG-carriers^12^ and important contributors to the infant resistance load. However, also Bacteroidales have been described as a potential ARG reservoir in the gut.^13^ Infant gut microbiota has been shown to harbor a larger proportion of ARGs than the adult population,^5,9^ even without prior antibiotic exposure,^3^ and only around 30% of the ARGs seem to be shared with the mother.^9^ As the infant gut microbiota transitions to a more adult-like composition during the first year of life, the relative proportion of ARGs in the infants’ gut has been reported to diminish.^5,14,15^ However, most previous infant resistome studies included only dozens of infants and a few time points, so the more robust developmental resistome trajectory is still unclear.

Previous studies have observed tetracycline resistance genes to be both prevalent and abundant resistance class in infants^6,16–18^ and adults.^2^ Nonetheless, the types of tetracycline resistance genes differ by age.^2^ Genes encoding resistance to beta-lactam,^6,16,18^ fluoroquinolone (quinolone),^6^ macrolides^6,16^ have also been reported as prevalent and abundant ARG classes, depending on the age of sampled infants. There appears to be major geographical differences in the proportions and types of ARGs found in humans around the globe due to differences in antibiotic use practices.^19,20^ Though, there is little variation in the most important taxa carrying specific ARGs globally, for example, the most common carbapenemase gene in the human gut, *cfiA*, is mostly found in *Bacteroides*.^21^ Still, more detailed information on the more specific resistome composition and its associations with the dynamics of the early life microbiota development and the related exposures is lacking.

We set to study the temporal development of the early life gut resistome in 475 infants from the Finnish Health and Early Life Microbiota (HELMi) cohort,^22^ based on four fecal samples from 3 to 24 months of age, and to characterize how different early life exposures and microbiome development affect this trajectory. Additionally, more than 300 maternal and 100 paternal samples were available to examine the within-family transmission of ARGs. We exploited the metagenomes of these 2,328 fecal samples to link both differences in exposures and gut microbiota composition to the resistome trajectory.

## Materials and methods

### Sample and data collection

The current study included 475 children from the HELMi cohort,^22^ for whom four high quality metagenome sequences derived from longitudinal fecal samples from the child’s first two years were available, more precisely at 3, 6, 12, and 24 months. Term singleton babies with a birthweight exceeding 2.5 kg and without known congenital defects were included in the cohort. The cohort consisted mostly of families living in the Helsinki metropolitan region and was recruited in 2016–2018. Additionally, 305 fecal metagenomes from the respective mothers and 123 samples from the fathers, sampled close to the delivery, were available. The parents collected the fecal samples and immediately stored them in −20°C, transported to the lab in frozen form where the samples were stored at −80°C until extraction. Information on the family’s lifestyle, children’s health, well-being and diet was collected with repeating questionnaires before the delivery (background) to more detailed questionnaires up to the age of two. Details related to delivery were collected from the hospital records. The full HELMi cohort has been previously described in detail.^22,23^ The study was approved by The Hospital District of Helsinki and Uusimaa (263/13/03/03 2015) and performed in accordance with the principles of the Helsinki Declaration. Parents signed an informed consent at enrollment.

### DNA extraction and sequencing

The DNA extraction was performed as previously described utilizing a bead-beating method.^23,24^ Briefly, approximately 250 or 340 mg of the fecal sample was suspended in 0.5 or 1 ml of sterile ice-cold PBS, and 250 μl of the suspension was combined with 340 μl of RBB lysis buffer (500 mM NaCl, 50 mM Tris-HCl (pH 8.0), 50 mM EDTA, 4% SDS) in a bead-beating tube from the Ambion MagMAX™ Total Nucleic Acid Isolation Kit (Life Technologies) for a repeated bead-beating step. Thereafter, 200 μl of the supernatant was used for DNA extraction utilizing KingFisher™ Flex automated purification system (Thermo Fisher Scientific) using MagMAX™ Pathogen High Volume protocol. DNA was quantified using Quant-iT™ Pico Green dsDNA Assay (Invitrogen).

Metagenomic sequencing was carried out using MGI technology. Library preparation and circularization of equimolarly pooled libraries was carried out with MGIEasy FS DNA Library Prep Set (MGI Tech, Shenzhen, China) and sequenced using DNBSEQ-G400RS High-throughput Sequencing Set (FCL PE150) according to manufacturer’s instructions (MGI Tech, Shenzhen, China).

### Taxonomic profiling and annotation of resistance genes

Quality filtering and removal of human sequences were performed on the FASTQ files using fastqc v.0.11.9^25^ and trimGalore v.0.6.6^26^ with default parameters. Quality-filtered sequences were screened to remove human sequences using Bowtie2 v.2.4.2^27^ against a non-redundant version of the Genome Reference Consortium Human Build 38, patch release 13 (available at https://www.ncbi.nlm.nih.gov/assembly/GCF_000001405.39/). After quality control and human read filtering, all early samples (3 and 6 months) with more than 5 million pairs of reads, late samples (12 and 24 months) with more than 10 million pairs of reads, and parental samples with more than 10 million pairs of reads were kept in the analysis. In total, 121 parental samples were excluded from the study for shallow sequencing, and 428 adult samples were kept in the analysis.

The sequences after quality and human filtering are openly accessible at the European Nucleotide Archive, under the project PRJEB70237. The detailed list of the samples used for this study and their accession codes is available in Supplementary file 1.

Taxonomic profiling was performed on reads after quality control using Kraken2^28^ and Bracken^29^ against the HumGut database.^30^ Taxonomic profiles were denoised using a prevalence threshold of 1% and a minimum abundance threshold of 0.001%. Fecal community types were determined by hierarchical clustering approach, using the hclust function in R and Ward’s minimum variance method^31,32^ on the family level aggregated taxonomic profiles using Aitchison distance. The distance method was chosen to limit the weight of single dominant taxa, issue present especially in the early, lower diversity samples. The number of community types were determined using the silhouette method.

To characterize the fecal resistomes, we mapped the reads with Bowtie2^27^ against the ResFinder4 database^33^ with the following options “-D 20 -R 3 -N 1 -L 20 -i S,1,0 5”. ResFinder4 includes clinically relevant and taxa-nonspecific ARGs that can be vertically and horizontally transferred (acquired resistance).

### Statistical analysis

The statistical analysis steps were performed with the statistical language R v.4.3.2.^34^ The antibiotic resistance gene tables were normalized with both the total sequencing depth and the gene size (reads per kilobase per million reads (RPKM)). Both taxonomic and ARG beta-diversity was calculated with the vegan package v.2.6–4^35^ utilizing Jaccard (binary) and Bray-Curtis dissimilarities. Jaccard was the primary method used for ARG profiles as the primary interest was in the presence or absence (carriage) of individual genes, whereas Bray-Curtis was used for the taxonomic profiles to account also for the relative abundances. ARG load was calculated by summing up the total RPKM normalized reads together by sample. To reduce noise in diversity estimations caused by sporadic mapping of short reads to ARGs with high sequence similarity over the length of the gene,^36^ the *bla* gene variants (*e.g*., *bla*_OXA-48_ and *bla*_OXA-58_) were aggregated based to the type (*e.g*., to TEM, SHV, or OXA), reducing the number of unique *bla* genes from 1517 to 41. Paired Wilcoxon test was used to compare the diversity measures over time, and Kruskal-Wallis coupled with Dunn’s test for multiple comparisons to compare groups within a time point. The Pearson’s Chi-squared test ($\chi^{2}$) was used to compare between categorical variables. The differential abundance of antibiotic resistance genes and gene classes was tested using DESeq2,^37^ negative binomial general linear model (GLM) adjusted for sequencing batch and infant identity when relevant, using Wald’s test to calculate the *p*-values. To estimate the ARG loads, the RPKM normalized counts were multiplied by 10,000 and rounded to integers for the negative binomial GLMs. A pseudocount of 1 was added to allow the comparison between detected and undetected genes. The multiplier was selected based on the RPKM count of the lowest detected ARG of 0.0017, to differentiate between the detected (17 after normalization) and undetected genes (1 after adding pseudocount).

ARG load was modeled with generalized linear models utilizing boosted GLM with the mboost package^38^ to select for the explanatory variables to remove variable redundancy and select the most predictive variables. The dataset was divided into a train (70%) and test sets (30%), and the final model was validated with the test set. ARG load and class-specific ARG loads were further correlated with the relative abundance of the six most abundant genera in the children’s samples with Spearman’s rank correlation.

Permutational multivariate analyses (PERMANOVA) were performed one sampling point at a time with the vegan function adonis2^35^ with 999 permutations, including the relevant variables based on previous literature and analyses to evaluate the effect of the different background variables on the overall ARG variation. The PERMANOVA models were adjusted for the sequencing batch. A backward selection for the best model was conducted using Akaike information criterion (AIC) for small sample sizes (2×k+n+ln(RSS/n) + (2×k×(k + 1))/(n−k − 1), where k = number or parameters, *n* = number of samples, RSS = residual sum of squares). A stricter criterion for reduction in AIC (1.5) was used to yield models with fewer variables.

The codes used for the main analyses submitted in https://github.com/rjokela/HELMi_resistome.

## Results

### Cohort overview

The study cohort comprised 475 infants, born in Finland in hospital at term, and their parent with available metagenome data (305 mother and 123 fathers). In total, 394 (83%) infants were vaginally delivered, and 81 (17%) were born by Caesarean delivery. Mothers of all Caesarean delivered infants and of 96 vaginally delivered infants received intrapartum antibiotics. Baseline characteristics, stratified by mode of delivery, are summarized in Table 1.

**Table 1.** Study cohort characteristics.

		Vaginal delivery	Caesarean delivery	All
	n	394	81	475
	Male infants, n (%)	202 (51%)	44 (54%)	246 (52%)
Maternal	BMI kg/m^2^ before pregnancy, median (IQR)	22.3 (20.6–24.4)	23.7 (22.0–26.2)	22.5 (20.7–24.8)
	Multiparous, n (%)	209 (53%)	37 (46%)	246 (52%)
	Bachelor level education or higher, n (%)	359 (91%)	69 (85%)	428 (90%)
	Gestational diabetes, n (%)	75 (19%)	17 (21%)	92 (19%)
Diet	Exclusive breastfeeding min. 3 months, n (%)	265 (67%)	37 (46%)	302 (64%)
	Any breastfeeding at 6 months, n (%)	366 (93%)	74 (91%)	440 (93%)
	Age at starting solids, weeks, median (IQR)	22 (18–28)	20 (18–22.5)	22 (18–24)
Antibiotics	Mother received antibiotics during pregnancy, n (%)	88 (22%)	21 (26%)	109 (23%)
	Intrapartum antibiotics, n (%)	96 (24%)	81 (100%)	177 (37%)
	Infant received antibiotics during 1^st^ year, n (%)	100 (25%)	22 (27%)	122 (26%)
	Infant received antibiotics by the end of 2 years, n (%)	198 (50%)	40 (49%)	238 (50%)
Parental samples	Maternal stool metagenome available, n (%)	251 (64%)	54 (67%)	305 (64%)
	Paternal stool metagenome available, n (%)	99 (25%)	24 (30%)	123 (29%)

BMI = Body mass index.

The gut microbiota and resistome composition was assessed based on shotgun metagenomic sequencing data derived from stool samples collected at 3, 6, 12, and 24 months from the children and parental samples close to delivery. After quality control and human read decontamination, the early infant samples (3 and 6 months) with more than 5 million pairs of reads and later samples (12 and 24 months) with more than 10 million pairs of reads were included (median = 44 × 10^6^ pairs, IQR = 15 × 10^6^). The lower cutoff for early samples was chosen due to the lower microbial biomass in these samples. For parental samples we used a cutoff of 10 million reads (median = 36 × 10^6^ pairs, IQR = 36 × 10^6^). A total of 2,328 samples, composed of 1900 infant samples and 428 parental samples were included.

### ARG dynamics and diversity

**Figure 1.**
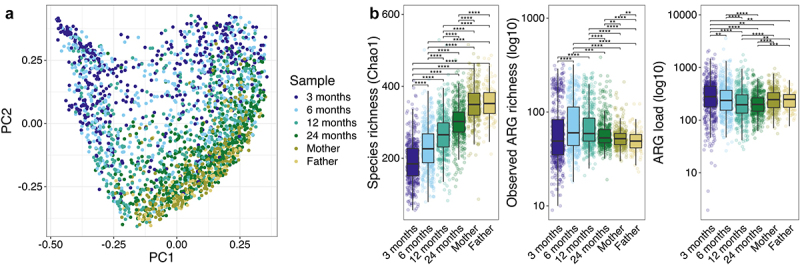
Resistome overview over the first two years and parental samples.

In total, 428 unique ARGs were identified with ResFinder4 in the whole dataset (Supplementary file 2). We observed a transition of the overall infant resistome composition toward parental resistome composition during the 2 years follow-up (Figure 1(a)), mirroring the maturation of the microbiota composition (Supplementary Figure S1).

a) Principal coordinate analysis of the Bray-Curtis dissimilarities of the antibiotic resistance gene compositions across the child samples and parental samples. b) Species richness, observed antibiotic resistance gene richness, and antibiotic resistance gene load by child age and parental samples. Paired and unpaired Wilcoxon test. *p < .05, **p < .01, ***p < .001 ****p < .0001.

While the bacterial richness increased with age, the trajectory of ARG richness and load was different (Figure 1(b)): The ARG richness increased from 3 months to 6 months (at the time when most infants had been introduced to solids), peaked between 6 months and 1 year, and decreased again at 2 years (*p* < .05, paired and unpaired Wilcoxon test). ARG richness was higher in 6 month and 1- and 2-year-old child samples than in the parental samples.

The ARG load decreased from 3 months to 1 year of age (*p* < .001, paired Wilcoxon test), and remained at a similar level between one and two years of age. ARG load was higher in 3-month-old infants compared to parents, but higher in parental samples compared to 1- and 2-year-old children. There was no statistical difference on the ARG loads between the mothers’ and fathers’ samples.

**Figure 2.**
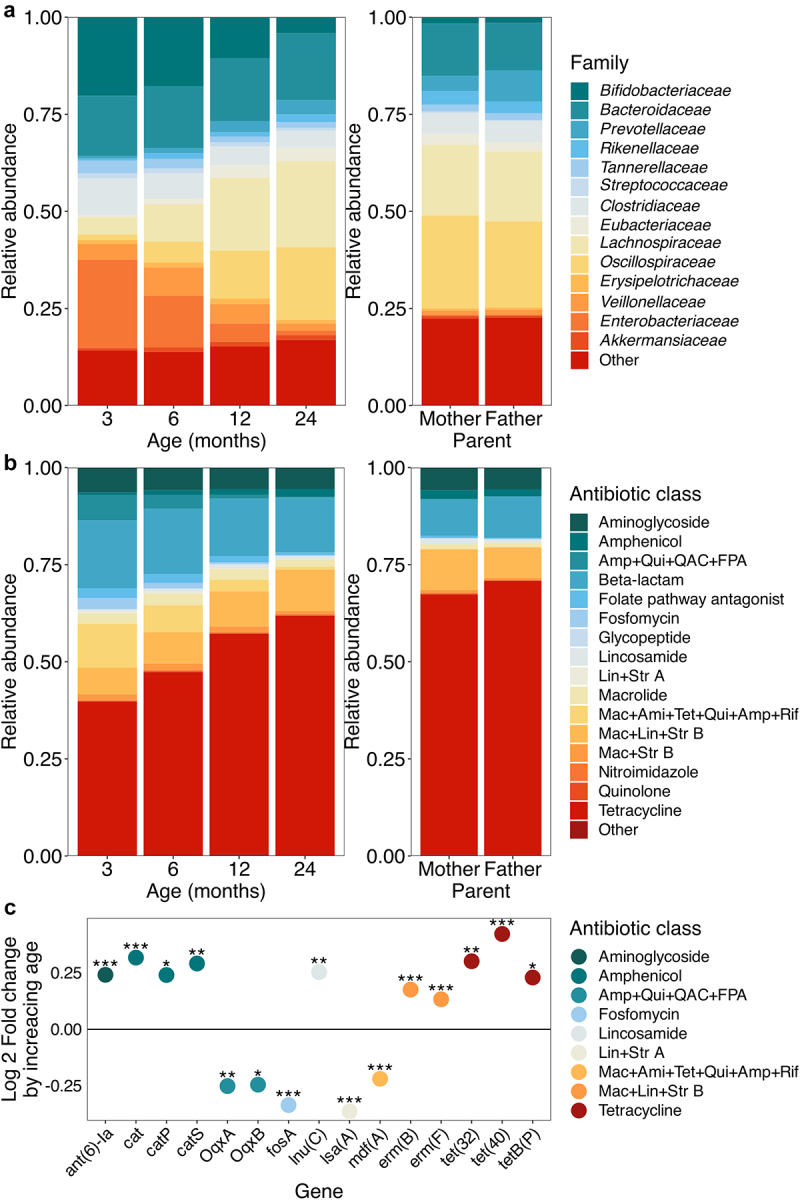
Taxonomic and resistome composition by age.

The resistome composition changed in parallel with the age-driven differences in the taxonomic composition (Figure 2). Tetracycline resistance was the resistance gene class with highest sum abundance in all infant samples and parental samples (50 unique genes detected), but generally represented a higher proportion in the parental samples (Figure 2(b)). The next most abundant classes of antibiotic resistance were beta-lactam (71 unique genes detected); macrolide-lincosamide-streptogramin B (11 unique *erm* genes detected); aminoglycoside; and macrolide-aminoglycoside-tetracycline-quinolone-amphenicol-rifamycin resistance (*mdf(A)* gene only).

Abundances of a) bacterial families and b) different antibiotic resistance classes in child samples across the 2 years and the parental samples. c) Antibiotic resistance genes differing significantly with child age in months. Positive values indicate increasing gene abundances by unit of age and negative values abundances decreasing by unit of age (used as a continuous variable in the model). The values were calculated with DESeq2 adjusting for the sequencing batch and infant identity, and *p*-values calculated with Wald’s test. Only *p*-values with a false discovery rate corrected *p*-value of < .1 are shown. *p < .05, **p < .01, ***p < .001. Ami = Aminoglycoside, Amp = Amphenicol, FPA = Folate pathway antagonist, Lin = Lincosamide, Mac = Macrolide, QAC = Quaternary ammonium compounds, Qui = Quinolone, Rif = Rifamycin, Str = Streptogramin, Tet = Tetracycline.

Abundances of ARGs classes varied with infant age. During the first two years of life, the antibiotic class amphenicol (DESeq2 adjusted for the sequencing batch and infant identity, Log2 fold change (FC) = 0.18, *p* = 4.5×10^−7^, Supplementary file 3) lincosamide (Log2FC = 0.27, *p* = .0043) and macrolide-lincosamide-streptogramin B (Log2FC = 0.065, *p* = .0074) resistances increased in abundance with increasing age. On the other hand, fosfomycin (LogFC = −0.35, *p* = 2.3×10^−4^), classes encoding resistance to lincosamide-streptogramin A (LogFC = −0.37, *p* = 1.0×10^−4^); macrolide-aminoglycoside-tetracycline-quinolone-amphenicol-rifamycin resistance (*mdf(A)* gene) (LogFC = −0.22, *p* = 1.8×10^−13^); and amphenicol-quinolone-quaternary ammonium compounds-folate pathway antagonist (*OqxA* and *OqxB* genes) (Log2FC = 0.–23, *p* = .015) decreased in abundance. Only 15 individual ARGs were found to differ statistically significantly with age, although the results were mostly consistent with the class-level observations (Figure 2(c)): three amphenicol resistance encoding genes, *cat*, *catP* and *catS* and lincosamide resistance encoding *lnu(C)* increased with age, and lincosamide-streptogramin A encoding *lsa(A)*, *mdf(A)*, and both *OqxA* and *OqxB* genes decreased with age. In addition, we saw an increase in the aminoglycoside resistance encoding *ant(6)-la*, macrolide-lincosamide-streptogramin B resistance encoding *erm(B)* and *erm(F)* genes, and tetracycline resistance genes *tet(32)*, *tet(40),* and *tetB(P)* and a decrease in fosfomycin resistance encoding *fosA*, not seen on the class level.

Mothers’ samples were collected around delivery and the samples collected before and after delivery were not significantly distinct in terms of ARG composition (PERMANOVA, *p* > .05, permutations = 999), nor in terms of ARG load, diversity or richness (*p* > .05, paired Wilcoxon test). Few genes involved in aminoglycoside, tetracycline and beta-lactam resistance were decreased in abundance in samples collected after delivery (Supplementary file 3).

### ARG sharing between family members

**Figure 3.**
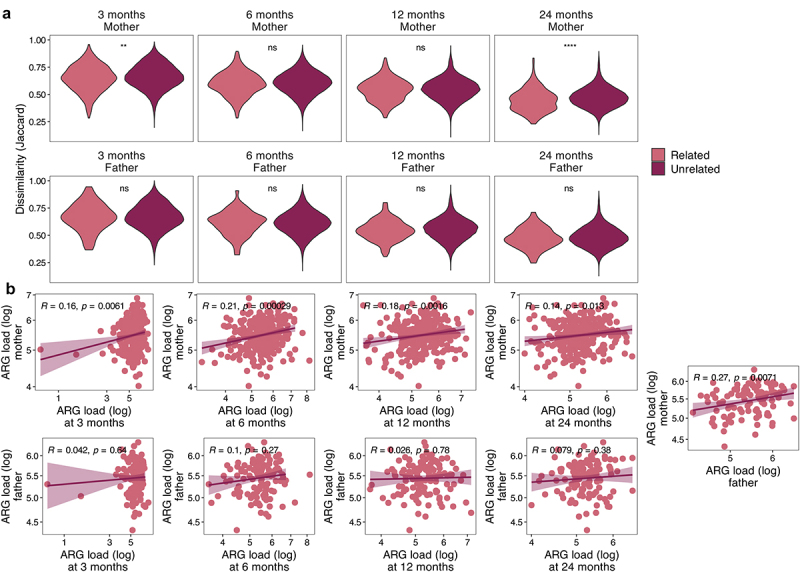
Resistome within the family unit.

The child resistome composition was significantly more similar to their own resistome over time than to other infants’ resistomes at any time point (*p* < .05, Jaccard distance, Wilcoxon test). Child’s resistome was also more similar to their own mother’s resistome compared to other mothers at 3 and 24 months (*p* < .05, Jaccard distance, Wilcoxon test) (Figure 3(a)). Interestingly, the infant resistome was not more similar to that of their own father than of unrelated fathers at any age (*p* > .05, Wilcoxon test). Additionally, the resistomes of the two parents from the same family were not significantly more similar to each other than to other parents (*p* > .05, Wilcoxon test). The pattern was similar in the bacterial community composition similarity within family members (Bray-Curtis dissimilarity on species level); children’s samples resembled their own mothers’ samples more than unrelated mothers’ only at 24 months (*p* < .0001), while father-infant pairs were not more similar than between-family pairs at any age (Supplementary Figure S2). No significant associations were found using Jaccard distance. Despite the varying within-family similarity on the resistome composition, the overall ARG load correlated between child and their own mother at all ages (Pearson’s correlation, Figure 3(b)), but never between child and father. However, the ARG loads correlated between the two parents (Figure 3(b)).

a) Density plots of Jaccard distances compared between the family members and unrelated individuals (*e.g*., child at 3 months and mother). The y axis ranges from 0 (identical) to 1 (no overlap). b) Antibiotic resistance gene load correlations between family members. Significance calculated using ANOVA (A) or Pearson’s correlation (B). *p < .05, **p < .01, ***p < .001.

### Taxonomic composition and background variables in relation to ARG composition

**Figure 4.**
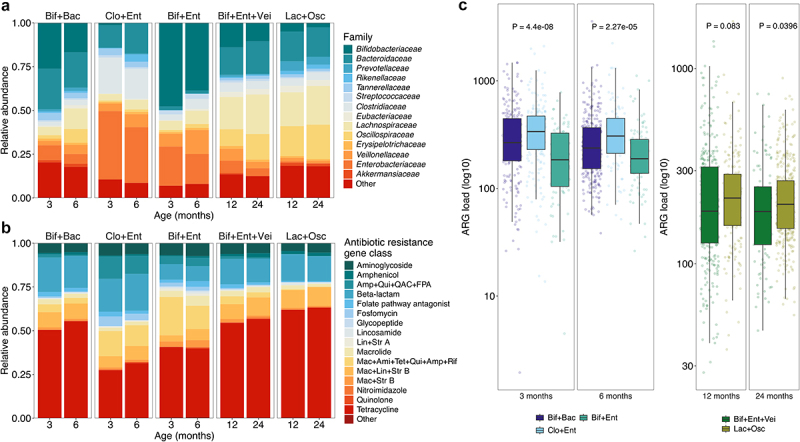
Early life bacterial community types and resistome.

To simplify the bacterial compositions, we performed hierarchical clustering of family level compositions with the Aitchison distance to identify different community types. In this unsupervised analysis, the 3- and 6-months samples were clustered together and represented three distinct community types (Figure 4(a), and Supplementary Figures S3a,c), and the latter two time points, 12 and 24 months, clustered into two separate community types (Figure 4(a), and Supplementary Figures S3b,c) named according to the dominant bacterial families Bif+Bac (Bif = *Bifidobacteriaceae*, Bac = *Bacteroidaceae*), Clo+Ent (Clo = *Clostridiaceae*, Ent = *Enterobacteriaceae*), and Bif+Ent in the earlier time points, and Bif+Ent+Vei (Vei = *Veillonellaceae*) and Lac+Osc (Lac =*Lachnospiraceae*, Osc = *Oscillospiraceae*) in the later time points. The early community types, but not the later, correlated with delivery mode, with most vaginally delivered infants found in the Bif+Bac community type and most Caesarean delivered in the Clo+Ent, especially at 3 months (Supplementary Table S1). The same phenomenon was seen relating to intrapartum antibiotic exposure, with non-exposed infants overrepresented in the Bif+Bac community type. Interestingly, children with older siblings (mother multiparous) ended more likely in the Bif+Bac community type than firstborn children (mother nulliparous before delivery) at 3 months, and at 24 months, were more often in the Lac+Osc community type. The ARG load differed by the bacterial community type, especially in the early communities with the Bif+Ent displaying the lowest load and Clo+Ent the highest (Figure 4(c) and Table 2, Dunn’s test for multiple comparisons *p* < .05, all comparisons). The differences were more modest in the later time points where Lac+Osc had slightly higher ARG load, but statistical significance was reached only at 24 months (Figure 4(c)).

a) Family level relative abundances of the bacterial taxa and b) relative abundances of the antibiotic resistance classes in different community types across the 2 years. c) Antibiotic resistance gene loads in the different community types across the 2 years. The community types in early (≤6 months) and late samples (≥12 months) are named based on the characterizing prevalent bacterial families. Values presented are false discovery rate adjusted *p*-values calculated with the Kruskal-Wallis rank sum test. Ami = Aminoglycoside, Amp = Amphenicol, FPA = Folate pathway antagonist, Lin = Lincosamide, Mac = Macrolide, QAC = Quaternary ammonium compounds, Qui = Quinolone, Rif = Rifamycin, Str = Streptogramin, Tet = Tetracycline. Bac = *Bacteroidaceae*, Bif = *Bifidobacteriaceae*, Clo = *Clostridiaceae*, Ent = *Enterobacteriaceae*, Lac = *Lachnospiraceae*, Osc = *Oscillospiraceae*, Vei = *Veillonellaceae*.

**Table 2.** The effect of selected variables on antibiotic resistance gene (ARG) composition and load.

Variable	Measure	3 months	6 months	12 months	24 months
Community type	Overall composition: R^2^	9.1%	9.8%	2.5%	1.6%
	*p*	< .001	< .001	< .001	< .001
	ARG load (RPKM): *p*	4.4 × 10^−8^	2.3 × 10^−5^	0.083	0.040
Delivery mode	Overall composition: R^2^	2.4%	1.1%	0.41%	0.3%
	*p*	< .001	< .001	.024	.12
	ARG load (RPKM): *p*	.13	.13	.35	.023
Intrapartum antibiotics in vaginal delivery	Overall composition: R^2^	2.4%	3.5%	2.6%	1.7%
	*p*	.24	.016	.18	.74
	ARG load (RPKM): *p*	.034	.23	.10	.56
Post-natal antibiotics	Overall composition: R^2^	0.20%	0.13%	0.17%	0.27%
	*p*	.46	.91	.66	.18
	ARG load (RPKM): *p*	.084	.19	.50	.012

Time point-wise permutational multivariate analysis results displayed for overall composition and Kruskal-Wallis results for ARG load except for post-natal antibiotics where Spearman’s rank sum correlation was used. R^2^ = coefficient of determination. RPKM = reads per kilobase per million reads.

To evaluate the effect of the community types in relation to different background variables on the overall ARG variation, we performed PERMANOVAs by time point, including the relevant variables based on previous literature and analyses and adjusting with the sequencing batch. After model selection, overall explanatory power of the variables remained modest (3 months: 17.2%, 6 months: 16.3%, 12 months: 8.2%, 24 months 7.9%) and the bacterial community type and diversity explained the largest share of the variation (community type: 1.4–8.7%, Chao1: 1.4–2.3%, *p* < .001) (Supplementary file 4). When analyzed alone adjusted for the sequencing batch, the community type explained almost 10% of the variation at the early time points and 1–2% at later ages (Table 2).

Apart from the microbiota variables, 4.0–9.0% of the ARG variation was explained by the background variables together, the significant variables in the time point specific models generally relating to delivery mode, parity, feeding, and post-natal antibiotics (Supplementary file 4). The delivery hospital was not associated with the composition of the infant resistome. As this study cohort was highly breastfed and had homogenous feeding patterns, we focused on the other variables in the subsequent analyses. The overall effect of formula feeding was modest (highest R^2^ in age-wise PERMANOVA adjusted for sequencing batch: 0.40%, *p* = .024 at 12 months).

We further examined the effect of the most important variables on the different ARG classes and genes with DESeq2 adjusted for the sequencing batch. The highest number of differentially abundant antibiotic resistance genes were found when comparing the bacterial community types (Supplementary Figure S4, Supplementary file 3) and many genes with age association (Figure 2(c)), associated also with the community types. For example, the *Enterobacteriaceae* associated *mdf(A)* gene was increased in both Clo+Ent and Bif+Ent compared to Bif+Bac and increased in Bif+Ent+Vei compared to Lac+Osc. Several *fosA* genes followed the same pattern. *Oqx* genes were elevated in the Clo+Ent community type compared to the two other community types, and lowest on Bif+Ent in the early time points, and lower in Lac+Osc compared to Bif+Ent+Vei later on. The genes associated with older age, (*e.g*., *vanHDX*, *aph(3’)-III*, and *tet(40)*), were generally increased in the Bif+Bac and Bif+Ent community types compared to the Clo+Ent community type.

### Resistome differences relating to delivery mode and early antibiotic exposure

**Figure 5.**
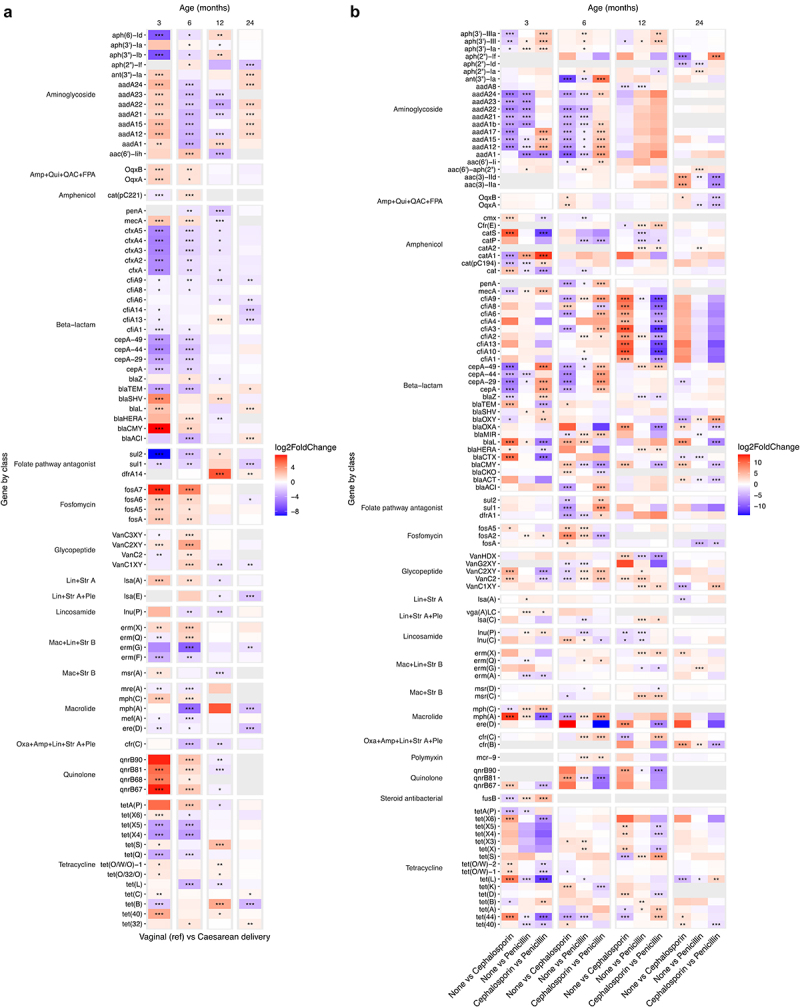
Differentially abundant antibiotic resistance genes between a) vaginal (reference) and Caesarean delivery and b) no intrapartum antibiotic exposure and the different antibiotic substances in vaginally delivered children.

Apart from the bacterial community variables only few lifestyle and exposure related variables stood out in the age-wise PERMANOVAs, mainly delivery and antibiotic exposure related variables, that we explored further. ARG load differed by delivery mode statistically significantly only at 24 months (Table 2), although infants born via vaginal delivery had a slightly higher average ARG load at all ages. Delivery mode associated with the overall ARG composition especially in the earlier time points (Table 2). The effect of delivery mode on the individual ARGs was the strongest and clearest in the two earliest time points (Figure 5(a), DESeq2, Supplementary file 3). Most notably, several fosfomycin resistance genes (*fosA* genes) and quinolone resistance genes (*qnrB* genes, encode ciprofloxacin resistance) were increased in Caesarean delivered children at 3 and 6 months but not later. By large, *tet(X)* genes (encode doxycycline, tetracycline, minocycline, and tigecycline resistance) and *tet(Q)* (doxycycline, tetracycline and minocycline) tended to appear in lower abundances in Caesarean delivered infants compared to vaginally delivered. In terms of beta-lactam resistance, mainly *cfxA* (antimicrobial susceptibility unclear), *cepA* (antimicrobial susceptibility unclear), *bla*_TEM_ (extended spectrum beta-lactamase (ESBL)) appeared in lower abundance in the Caesarean delivered infants, while most of the remaining *bla* genes, most importantly *bla*_SHV_ and *bla*_PLA_ (both ESBLs), were elevated in Caesarean delivered compared to vaginally delivered. Macrolide resistance genes appeared mostly in lower numbers in Caesarean delivered 3–6-years-olds compared to vaginally delivered, except for *mph(A)* that was observed in higher levels in vaginally delivered at 3 and 6 months compared to Caesarean delivered. Interestingly, *ere(D)* and *mph(A)* were still found in lower levels in Caesarean delivered children at 24 months. Most early differences in the ARG abundances seemed to relate more to the delivery mode itself than the differences in intrapartum antibiotic exposure between the delivery modes, as there was a difference in Caesarean delivery when compared both to vaginal delivery with and without antibiotics (Supplementary Figure S5a, Supplementary file 3).

The values were calculated utilizing DESeq2 negative binomial general linear models adjusting for the sequencing batch, and *p*-values calculated with Wald’s test. Only genes with at least two significant results are displayed. Only *p*-values with a false discovery rate corrected *p*-value of < .1 are shown. *p < .05, **p < .01, ***p < .001. Children, whose mothers received other antibiotics, or a combination of antibiotics (*N* = 15) were excluded from this analysis. N_none_ = 298, N_cephalosporin_ = 18, N_penicillin_ = 63. Amp = Amphenicol, FPA = Folate pathway antagonist, Lin = Lincosamide, Mac = Macrolide, Oxa = Oxazolidinone, Ple = Pleuromutilin, QAC = Quaternary ammonium compounds, Qui = Quinolone, Rif = Rifamycin, Str = Streptogramin, Tet = Tetracycline.

In terms of intrapartum antibiotics, the effect depended on the choice of antibiotic. We examined differences between antibiotic substances in the vaginally delivered infants, and the effect on both ARG load and overall ARG composition was low and reached statistical significance only at 6 months for the composition and 3 months for the load (Table 2). No difference between penicillin and cephalosporin exposed infants was observed in ARG load (Dunn’s test *p* = .90). Although compared to no exposure, the effects of penicillin and cephalosporin were somewhat similar, *aadA* genes were more abundant in penicillin-exposed than cephalosporin exposed at 3 and 6 months (Figure 5(b), Supplementary file 3). Some class A beta-lactamases putatively able to hydrolyze cephalosporins (*e.g*., *bla*_CTX_, *bla*_L_, *bla*_TEM_) were mainly elevated in cephalosporin exposed. At 12 months, *cfiA* genes (metallo-beta-lactamase that confers resistance to cephalosporin^39,40^) were elevated in the cephalosporin exposed, but not in penicillin exposed, but the effect was no longer significant at 24 months.

The effect of infants’ post-natal antibiotic history on individual ARGs was also strongest in the two earlier time points (Supplementary Figure S5b, Supplementary file 3). Most notably, aminoglycoside resistance genes (*aph* ang *aadA* genes) were decreased, and beta-lactam resistance genes (*cfxA* and different *bla* genes) were increased with increased antibiotic exposure. There were fewer significantly differing gene abundances at 12 and 24 months, but *aadA* genes showed a slight increase (non-significant) and several *bla* genes decreased, especially at 12 months with increased antibiotic exposure. No associations with the overall ARG composition nor the ARG load were observed (Table 2).

### The role of individual genera and species on the early resistome

Next, we aimed to compare the weight of individual genera compared to early life exposures linked to the intestinal resistance load at the ages between 3 and 24 months with boosted general linear models that selects the variables most linked with the ARG load and removes the collinear variables. The models were validated with a training and a test set of the data. The analysis was performed by time point with the background variables and the rare-filtered relative abundances of bacterial genera, and with only the background variables. Overall, the models including the bacterial genera performed considerably better, explaining 13% (root mean square error (RMSE) = 254), 8.1% (RMSE = 287), 27% (RMSE = 172), and 28% (RMSE = 119) of the ARG load in the test set at 3, 6, 12, and 24 months, respectively (Supplementary file 5), whereas the background variables alone explained a maximum of 0.5%. Therefore, we focused on the former.

**Figure 6.**
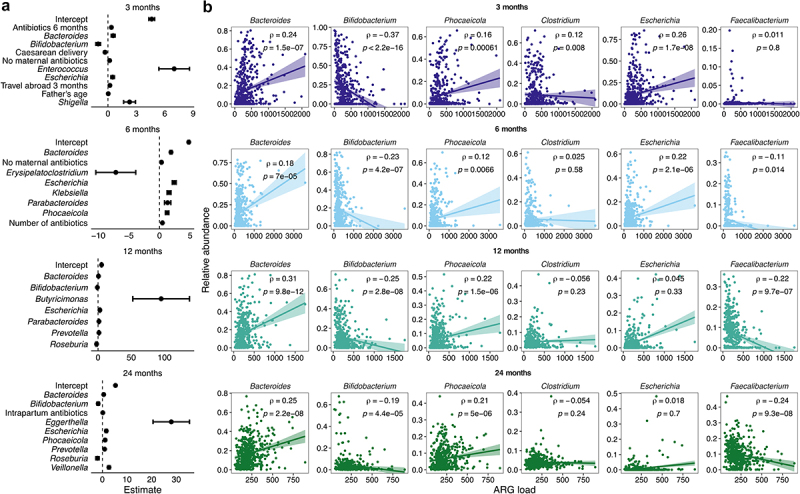
Antibiotic resistance gene load in relation to bacterial genera and early life exposures.

Relative abundances of *Bacteroides, Escherichia*, and *Prevotella* spp. were linked to increased ARG load in several time points, whereas the relative abundances of *Bifidobacterium*, *Faecalibacterium*, and *Roseburia* spp. were associated with lower ARG load (Figure 6(a), Supplementary file 5). Additionally, pathobionts like *Klebsiella* and *Shigella* were associated with higher ARG load in the neonatal samples. Counterintuitively, lack of exposure to maternal antibiotics during pregnancy predicted a higher ARG load at 3 and 6 months. The addition of the bacterial genera overrode the effect of most background variables, but most of the remaining variables described infant antibiotic history, mostly increasing the ARG load. Interestingly, in the models without genera, Caesarean delivery predicted a lower ARG load in all ages and formula use at all 6- and 12-month models (Supplementary file 5). We repeated the analysis on the parental samples and found partly the same genera associating with ARG load especially in mothers, most importantly *Bacteroides* and *Phocaeicola* correlating with higher load and *Bifidobacterium* with a lower load (Supplementary file 5, Supplementary Figure S6a).

a) Generalised linear models explaining the antibiotic resistance load by age. Only the significant (*p* < .05) variables are presented, and whiskers mark standard error. Estimate defines the coefficient used to predict the final load and the final effect size for the bacterial genera is dependent on the relative abundance of each genus. b) Spearman’s rank correlations between antibiotic resistance load and the relative abundance of the 6 most abundant genera in child samples by age.

To confirm the findings from the generalized linear models, we explored the correlations of the six most abundant genera with the ARG load across the child samples (Figure 6(b)), and separately for the five most abundant ARG class loads: 1) tetracycline; 2) beta-lactam; 3) macrolide-lincosamide-streptogramin B (*erm* genes only) B; 4) aminoglycoside; and 5) macrolide-aminoglycoside-tetracycline-quinolone-amphenicol-rifamycin (the *mdf(A)* gene only) (Supplementary Figures S7–11). *Bacteroides*, *Phocaeicola* (formerly part of the *Bacteroides* genus), and *Escherichia* correlated generally with a higher ARG load, whereas *Bifidobacterium* and *Faecalibacterium* correlated with a lower ARG load (Figure 6(b)). *Escherichia*-load correlation was significant only in the first two time points and *Faecalibacterium*-load correlation in the latter two. In parental samples, *Bifidobacterium* and *Faecalibacterium* were the genera with the strongest association with ARG load, both associated with lower ARG load (Supplementary Figure S6b, *r* = −0.24 to −0.41). Relative *Bacteroides* and *Phocaeicola* abundances correlated positively with tetracycline (Supplementary Figure S7), beta-lactam resistance gene (Supplementary Figure S8), and *erm* gene load (Supplementary Figure S9) in almost all time points, but not with aminoglycoside (Supplementary Figure S10) nor the *mdf(A)* gene (Supplementary Figure S11). Aminoglycoside resistance gene load correlated moderately (*r* = 0.14–0.19) with relative *Clostridium* abundance. The *mdf(A)* gene correlated mainly and strongly (*r* = 0.94–0.97) with relative *Escherichia* abundance. Relative *Bifidobacterium* abundance correlated negatively especially with early beta-lactam resistance gene load and later tetracycline resistance gene load at the later time points. *Faecalibacterium*, that also increased in prevalence and abundance with age, correlated negatively with the *mdf(A)* gene load and aminoglycoside resistance gene load.

Correlations between relative abundance of individual bacterial species and abundance of ARGs were performed on the child samples to examine deeper the potential ARG-carriers (Supplementary file 6). The strongest correlations (Pearson correlation coefficient (PCC)>0.6) were observed mostly in pathogens and pathobionts, such as *Escherichia coli* (*mdf(A)*), different *Klebsiella* species (*e.g*., *bla*_SHV_, *fosA* and *OqxA*), and *Proteus mirabilis* (*tet(J)*). Among commensals, *Bacteroides fragilis* showed strong correlations with four beta-lactam resistance encoding genes, *cepA* (PCC = 0.81), *cepA-29* (PCC = 0.56), *cepA-49* (PCC = 0.55), and *cepA-44* (PCC = 0.45).

## Discussion

In this study we utilized over 2,300 fecal metagenomes to explore the characteristics and dynamics of the intestinal resistome between 3 and 24 months of life, and how the parental resistome relates to the infant resistome. Our study extends the previous research on the vertical transmission of the resistome from mother-infant dyads to include also the father. Our results indicate that even though resistance genes are shared within family unit, father’s role in infant resistome appears negligible during the first two years of life. Interestingly, despite the ARG composition being only moderately shared between the family members, the ARG loads were correlated between mothers and children as well as between the spouses. As the ARG load correlates within family, it is possible that with a larger paternal sample set and maybe older children, also father-child ARG load correlation would be observable. Recently, father has been shown to be a stable source of bacterial strains alongside the mother during the first year of life,^41^ indicating also ARG transmission occurs between father and child, not observable with the distance methods used in this study.

Tetracycline was the most abundant resistance class across all ages in the children’s samples as well as in the parental samples, although tetracycline use in infants is rare and was previously avoided in children under 8 years-of-age.^42^ The most abundant antibiotic resistance classes identified in this study were mostly convergent with the earlier literature, with tetracycline^6,16–18^ and beta-lactams^6,16,18^ as the two most abundant classes. However, fluoroquinolone and other quinolone resistance genes were not observed in high abundances in our data set, but was the second most abundant resistance type reported by Li et al.^6^ in a Danish cohort of 1-year-old infants. Macrolide resistance genes and genes encoding multiresistance including macrolide resistance have been reported prevalent in early gut microbiota.^6,16–18^ In this dataset, multiresistance genes also encoding macrolide resistance (*e.g*., *erm* genes) were more common than simply macrolide resistance genes, but two of the five most common resistance gene classes included macrolide resistance. Macrolide use has been identified to increase resistance to said antibiotic^43^ and affect infant gut resistome composition longer than penicillin,^6^ have long-lasting effect on the infant gut microbiota composition,^44^ and is an important antibiotic in pediatric care.^45^

The resistome development during the two first years of life occurred parallelly with the gut microbiota development with community types and gut microbiota trajectories reflected in the resistome profile. While the typical infant gut colonizer *Bifidobacterium* was associated with lower ARG load, the other dominant early colonizer, *Bacteroides*, was a main taxon associated with higher ARG load. This was also seen in the early community types, where *Bacteroidaceae* and *Enterobacteriaceae* dominant community types had higher ARG loads, and the *Bifidobacteriaceae* dominant type had lower loads even at 24 months. Even adult samples that were dominated by other taxa, replicated these associations. High *Bifidobacterium* levels have previously been associated with low ARG abundance^46,47^ and the order Bacteroidales, has been identified as a potential reservoir for clinically relevant ARGs in the gut through invertible promoters regulating ARGs.^13^ *Bacteroides* spp. were recently identified as an important plasmid host in the infant gut microbiota, and a large share of horizontal gene transfer events occur in *Bacteroides*.^48^ Additionally, *Bacteroides cellulosilyticus* was identified as an important source of maternal microbiome genes transferring to other species in the infant microbiota.^10^ This suggests a potentially higher mobilization and dissemination of ARGs in the gut microbiota community dominated by *Bacteroides* and highlight the need of further investigation of the interaction between the mobilome and the resistome in infant gut microbial communities. Bacteroidetes phylum (Bacteroidota in the new nomenclature^49^) has also been found to have a high prevalence of tetracycline resistance^50^ and many beta-lactamase encoding genes, such as carbapenemase encoding *cfiA* that appears to be restricted to *Bacteroides*.^21^ Genes encoding tetracycline and beta-lactam resistance were the two most abundant resistance classes in this study and correlated with relative *Bacteroides* abundance.

Likewise, *Enterobacteriaceae*, usually found in higher proportion in the gut in early life than in adulthood, are important ARG carriers.^12^ *E. coli* has been identified in many previous studies as the main driver of the early gut resistome.^6,18,51,52^ Furthermore, a recent study showed that *Bacteroides* beta-lactamase genes could be transferred to *E. coli* in laboratory conditions.^21^ It should be noted that antibiotic resistance research has focused on the clinically relevant strains and laboratory strains, meaning genes from these strains are better covered in their reference libraries than the genes harbored by other taxa.^53^ The same bias was discovered by He *et al.* ^48^ who report *Bifidobacterium* and *Bacteroides* plasmids unrepresented in the database compared to the more well characterized *Escherichia* and *Klebsiella*. Still, the role of *Enterobacteriaceae* in terms of gut resistome is clear, and the family contains many clinically relevant taxa.

Conversely to previous literature,^5^ we found Caesarean delivery to be associated with a decreased ARG load, likely relating to the decreased levels of *Bacteroides* spp. in Caesarean delivered children,^54,55^ despite the higher abundance of pathobionts often carrying ARGs.^8^ The effect of birth mode on the resistome composition was strongest at 3 and 6 months, the higher abundance of *Bacteroides* spp. in vaginally delivered infants likely contributing to the higher abundance of the abundant tetracycline and beta-lactam resistance genes. However, *Bacteroides* abundance alone does not explain the large ARG load in vaginally delivered infants, and for example intrapartum antibiotics could play a role in the resistome development. Some studies report a higher ARG load in Caesarean delivered infants as late as 1 year of age, although statistical significance was not reached.^6^ The high breastfeeding rates even among Caesarean delivered infants in our study cohort likely promoted *Bifidobacterium* growth, thus reducing the overall resistance load,^56^ potentially mitigating the persistent Caesarean delivery-related microbiota and resistome effects documented in other cohorts.^5,6^ We did not find differences on infant gut resistome in relation to the birth hospital, but as the first sampling point in this study was at 3 months, we may have missed transient effects from earlier exposures. Additionally, the overall high breastfeeding rates homogenize the overall microbiota compositions between the different exposure groups.

Certain antibiotic resistance genes associated clearly with child age and the related bacterial community type. The *OqxA* gene that is found in the same operon as *OqxB*, characterized in many clinically relevant *Enterobacteriaceae*,^57^ were mainly found in the early samples at 3 and 6 months, and was associated with the bacterial community types driven by higher *Enterobacteriaceae* abundance. Fosfomycin resistance genes, especially *fosA* and *fosA5*, found in many clinically relevant Gram-negative bacteria,^58^ exhibited a similar pattern the *OqxA* in the resistome during the first 3–24 months of life. The multidrug resistance encoding *mdf(A)*, that was found to correlate strongly with *Escherichia*, characterized as an *E. coli* specific gene in the literature,^59^ was overall the fifth most abundant in terms of resistance classes, but also decreased in time and was more abundant in the *Enterobacteriaceae* driven bacterial community types compared to other community types. Interestingly none of these genes were elevated in the Bif+Bac community type that could be considered the least disrupted early gut microbiota composition.^5^ We found a higher number of ARGs to associate with increased age, even though the overall ARG load decreased. Many of the older-age-associated genes, such as *cat* and *catP* genes, encoding chloramphenicol resistance, appeared in higher abundance in the more adult-like Lac+Osc community type at 12 and 24 months. Overall, due to the major changes in the infant gut microbiota during the early development, it is important to consider the developmental stage of the microbiota when comparing resistome results between different studies and populations.

The bacterial community types were highly correlated with delivery mode. The bacterial community differential abundance results were somewhat converging with the delivery mode results in the early time points, with same genes reducing or increasing in Caesarean delivered infants compared to vaginally delivered and Clo+Ent and Bif+Ent community types compared to Bif+Bac. This was expected as the Bif+Ent and Clo+Ent community types were more common in Caesarean delivered infants than in vaginally delivered. The differentially abundant genes by delivery mode (*e.g*., *cfxA* and *fosA* genes, *bla*_TEM_, *Tet(Q)*, *OqxA* and *OqxB*) found in this study were largely similar than reported by a recent Australian study on 1 week old infants.^17^ Although the effect of the delivery mode was much stronger than the effect of intrapartum antibiotics (intrapartum antibiotics were administered in all Caesarean deliveries), the choice of antibiotic mattered in vaginally delivered infants exposed to intrapartum antibiotics. Cephalosporin exposure in children was associated with increase in putative cephalosporin hydrolyzing ARGs, even though the general ARG load was not impacted significantly.

We found no major associations between antibiotic exposure history and the resistome. The main differences on the individual gene level were observed during the first 6 months despite the number of antibiotic exposures and number of exposed infants increased with age. Although 74% of the antibiotic courses were penicillins, mostly amoxicillin (with or without clavulanic acid as beta lactamase inhibitor), the individual antibiotic exposure histories differed in timing. It is also worth noting that the antibiotic exposure rates reported in this cohort are lower than what has been reported in other European countries,^60,61^ and much lower than in low- and middle-income countries.^62^ Infants with prevalent and repeated antibiotic exposures might be more at risk to harbor a higher ARG load,^63^ not applicable in this dataset.

In this study cohort, *Bacteroides* spp. were common taxa associated with a non-perturbated microbiome. However, *Bacteroides* species are increasingly resistant to antibiotics though untargeted exposure to antibiotics as they are a classical human microbiome component especially in modern western populations. Tetracyclines are used commonly in production animals, and they were one of the first commercially available antibiotics, which could explain the abundance of tetracycline resistance genes in infants that had not been exposed to tetracycline. Of note, a commonly used probiotics such as *Bifidobacterium animalis* subsp. *lactis* also contain a tetracycline resistance gene,^64^ possibly also contributing to tetracycline resistance in early life. *Bacteroides* spp. has also been associated with health effects in humans,^65^ implying that the question of early resistome mitigation can be complicated.

Antibiotic resistance is part of most natural ecosystems^66^ and carried by many commensal bacteria, but human actions can affect the prevalence and abundance of these genes in our surroundings. The interaction between mobile genetic elements and antibiotic resistance genes is of particular interest as the mobilome may play a crucial role in the horizontal transfer and spread of antibiotic resistance across different bacterial species in the gut. However, the challenges of assembling ARGs within mobile genetic elements and accurately predicting their host bacterial species using short-read metagenomic sequencing data, is challenging. ARGs and mobile genetic elements often contain repetitive sequences and regions of high similarity, limiting the assembly quality in complex metagenomes, and can lead to overlooking low abundance of ARGs. Since it is difficult to capture the full genetic context of ARGs, disentangle their precise genomic locations, and reliably associate them with specific host species based solely on short-read metagenomic data, we purposefully limited our investigation to the broader associations with the microbiome composition and the resistome. This large longitudinal cohort shows the infant resistome is not only composed by the common pathogens, but also some key early colonizers and commensals such as *Bacteroides* spp. The antibiotic resistance databases often concentrate on genes from clinical isolates or common laboratory strains like *E. coli*, possibly skewing our view. Still, it is important to understand the dynamics of the resistome of one our most vulnerable populations and understand better how that resistome is connected with that of caregivers.

## Supplementary Material

Supplemental Material

## Data Availability

The raw sequencing data supporting this study are openly available at the European Nucleotide Archive, under the project PRJEB70237 (https://www.ebi.ac.uk/ena/browser/view/PRJEB70237), and the samples included in the study are detailed in the Supplementary Table 1.
