# Supplementary material for: A cohort study in family triads: impact of gut microbiota composition and early life exposures on intestinal resistome during the first two years of life
Source: Gut Microbes. 2024 Aug 2;16(1):2383746. doi: 10.1080/19490976.2024.2383746 (PMC11299627; doi:10.1080/19490976.2024.2383746)
Supplement: Supplemental Material [file KGMI_A_2383746_SM2027.zip › Supplementary_figures_and_tables.docx]

# Supplementary figures and tables

## Supplementary Figures:


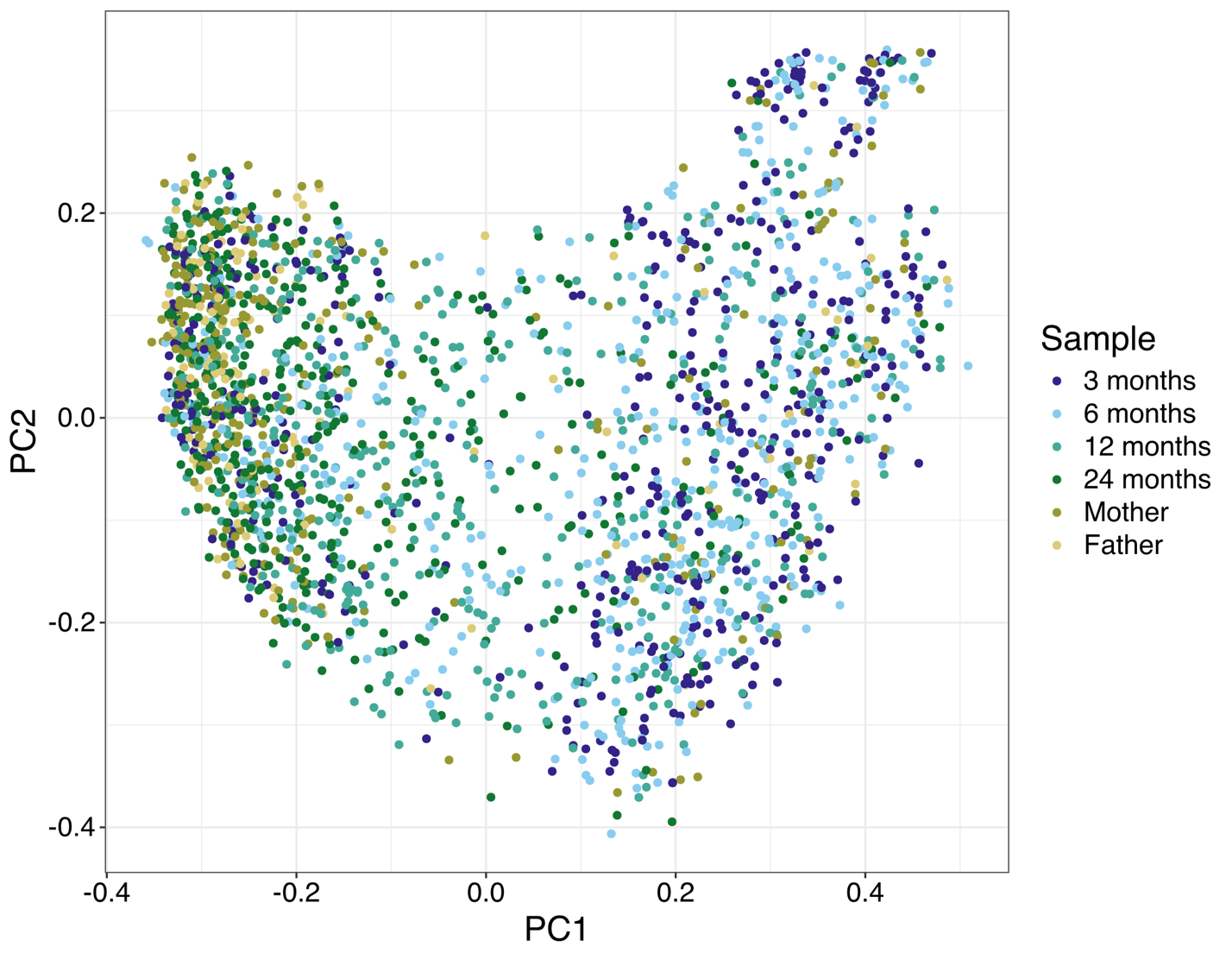


**Supplementary Figure S1** **Principal coordinates analysis plot of the relative species compositions in children and parents.** The distances were calculated with the Bray-Curtis dissimilarity index.


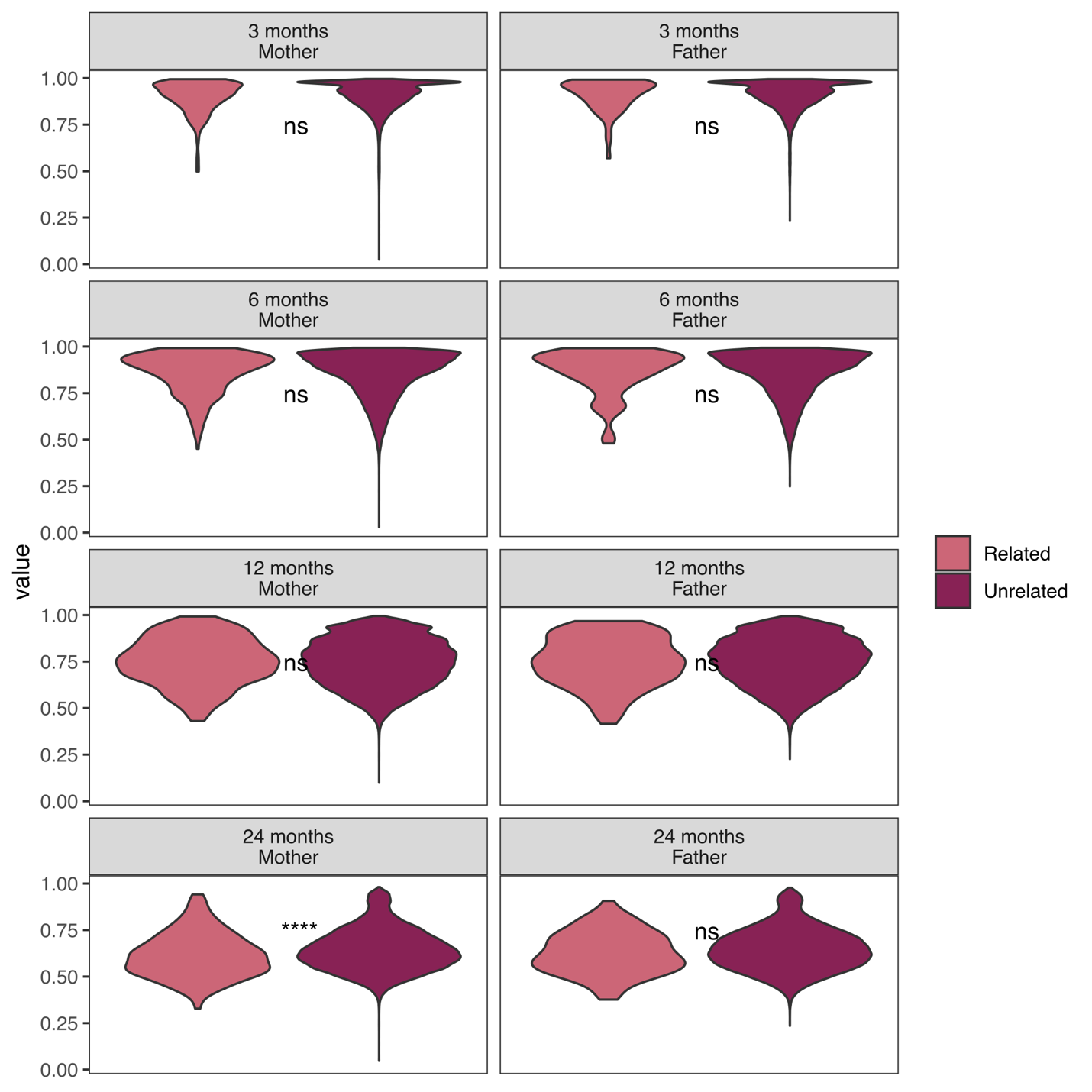


**Supplementary Figure S2** **Species transmission**. Density plots of Bray-Curtis dissimilarities compared between the same family unit and unrelated individuals. Significance calculated using ANOVA. The y axis ranges from 0 (identical) to 1 (no cross-over).


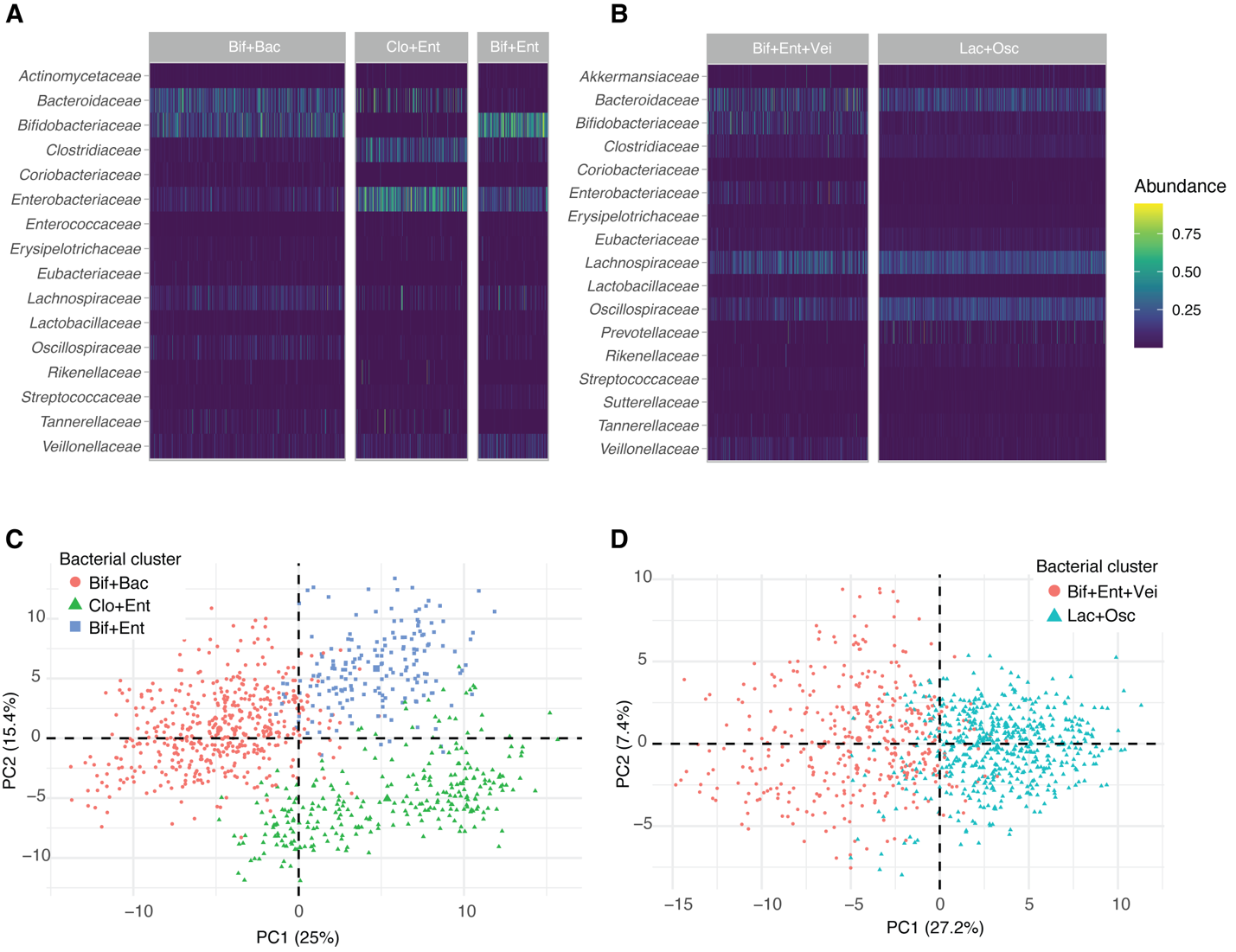


**Supplementary Figure S3 Family level community types in the infant gut.** Relative abundance of the most abundant bacterial families (families reaching a minimum abundance 1% and 10% prevalence) in samples divided in the bacterial community types A) at 3 and 6 months, and B) 12 and 24 months. Principal component analysis plot of the species-level composition based on Aitchison distance coloured by the bacterial community type C) at 3 and 6 months, and D) 12 and 24 months. Community type sizes by age: 3 months, N_Bif+Bac_=208, N_Clo+Ent_=185, N_Bif+Ent_=82; 6 months, N_Bif+Bac_=282, N_Clo+Ent_=97, N_Bif+Ent_=96; 12 months, N_Bif+Ent+Vei_=297, N_Lac+Osc_=178; 24 months, N_Bif+Ent+Vei_=97, N_Lac+Osc_=378. Bac = Bacteroidaceae, Bif = Bifidobacteriaceae, Clo = Clostridiaceae, Ent = Enterobacteriaceae, Lac = Lachnospiraceae, Osc = Oscillospiraceae, Vei = Veillonellaceae.


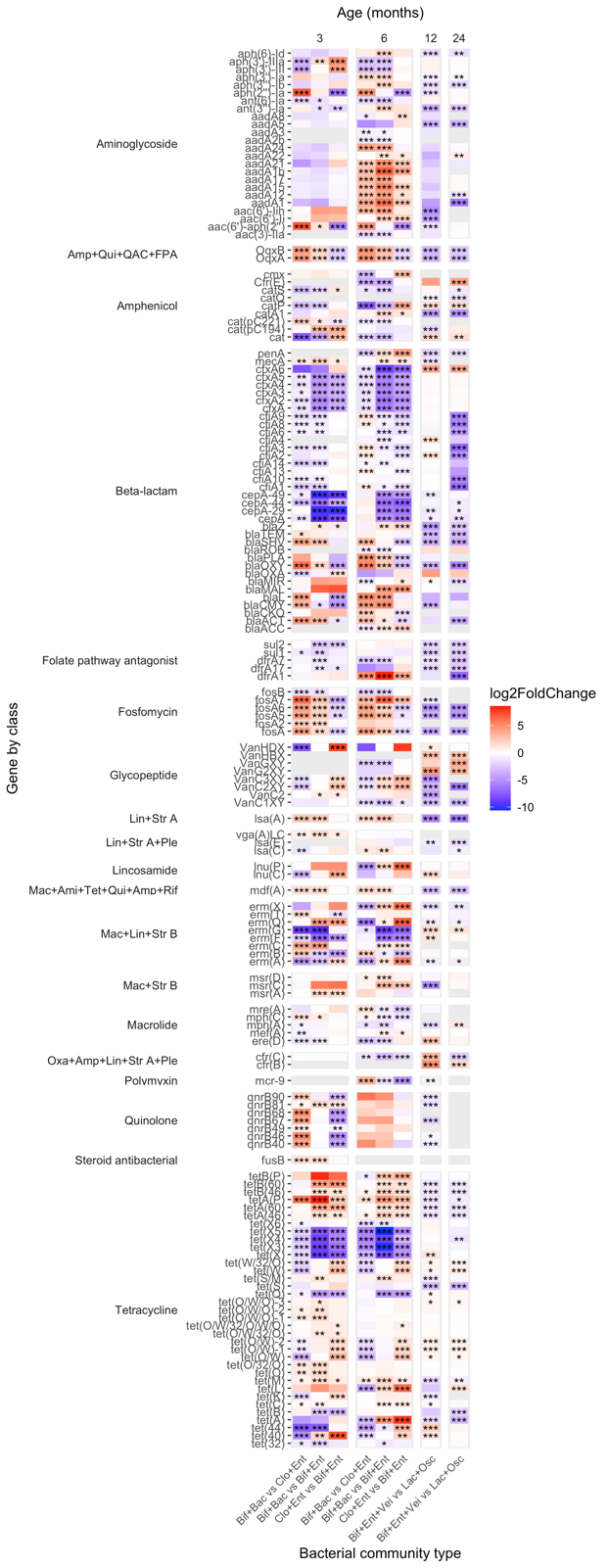


**Supplementary Figure S4 Differentially abundant antibiotic resistance genes between the different bacterial community types.** The reference variable is listed first. The values were calculated utilising DESeq2 negative binomial general linear models adjusting for the sequencing batch, and P-values calculated with Wald’s test. Only genes with at least two significant results are displayed. Only P-values with a false discovery rate corrected P-value of <0.1 are shown. *: P<0.05, **:P<0.01, ***:P<0.001. Amp = Amphenicol, FPA = Folate pathway antagonist, Lin = Lincosamide, Mac = Macrolide, Oxa = Oxazolidinone, Ple = Pleuromutilin, QAC = Quaternary Ammonium Compounds, Qui = Quinolone, Rif = Rifamycin, Str = Streptogramin, Tet = Tetracycline.


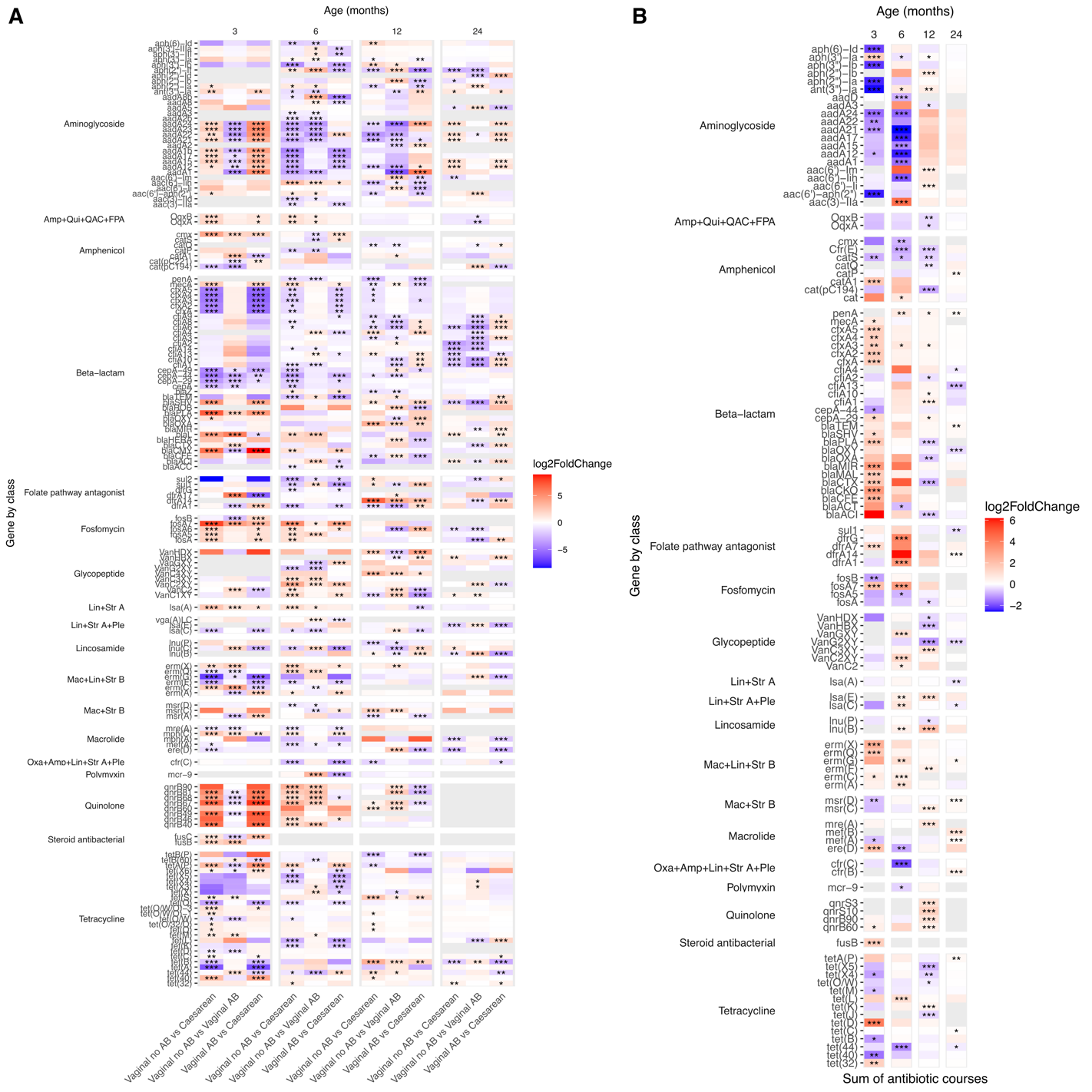


**Supplementary Figure S5 Differentially abundant antibiotic resistance genes A) between no intrapartum antibiotic exposure in vaginal delivery and the antibiotic exposed vaginally or Caesarean delivered children and with B) increasing antibiotic history at sampling time.** The reference variable is listed first. The values were calculated utilising DESeq2 negative binomial general linear models adjusting for the sequencing batch, and P-values calculated with Wald’s test. In A, only genes with at least two significant results are displayed, and for B, genes with at least one significant result are displayed. The sum of lifetime post-natal antibiotic courses by time point were used as a continuous variable in B, thus log2 fold change indicates change per change in unit of antibiotic exposure. Only P-values with a false discovery rate corrected P-value of <0.1 are shown. *: P<0.05, **:P<0.01, ***:P<0.001. Amp = Amphenicol, FPA = Folate pathway antagonist, Lin = Lincosamide, Mac = Macrolide, Oxa = Oxazolidinone, Ple = Pleuromutilin, QAC = Quaternary Ammonium Compounds, Qui = Quinolone, Rif = Rifamycin, Str = Streptogramin, Tet = Tetracycline.


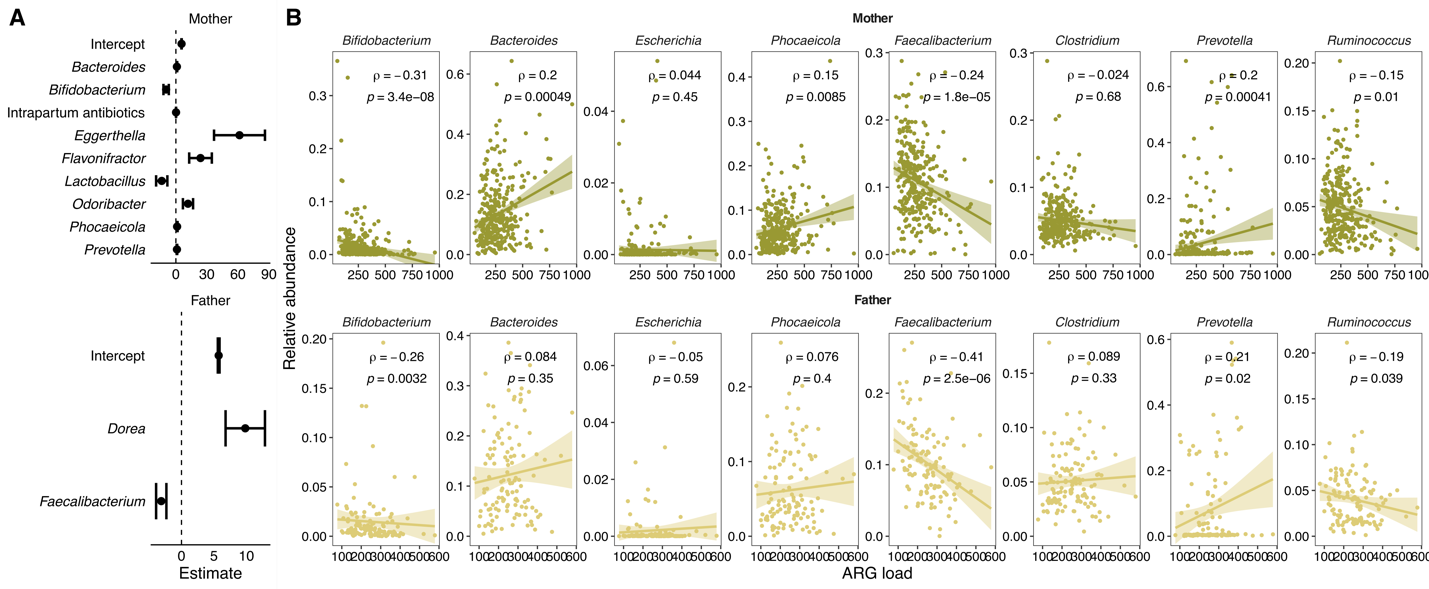


**Supplementary Figure S6 Antibiotic resistance gene load in relation to bacterial genera and exposures in the parental samples.** A) Generalised linear models explaining the antibiotic resistance load in mothers’ and fathers’ samples. Only the significant (P<0.05) variables are presented, and whiskers mark standard error. Estimate defines the coefficient used to predict the final load and the final effect size for the bacterial genera is dependent on the relative abundance of each genus. B) Spearman’s rank correlations between antibiotic resistance load and the relative abundance of the 6 most abundant genera, and genera presented in figure 4 in mothers’ and fathers’ samples.


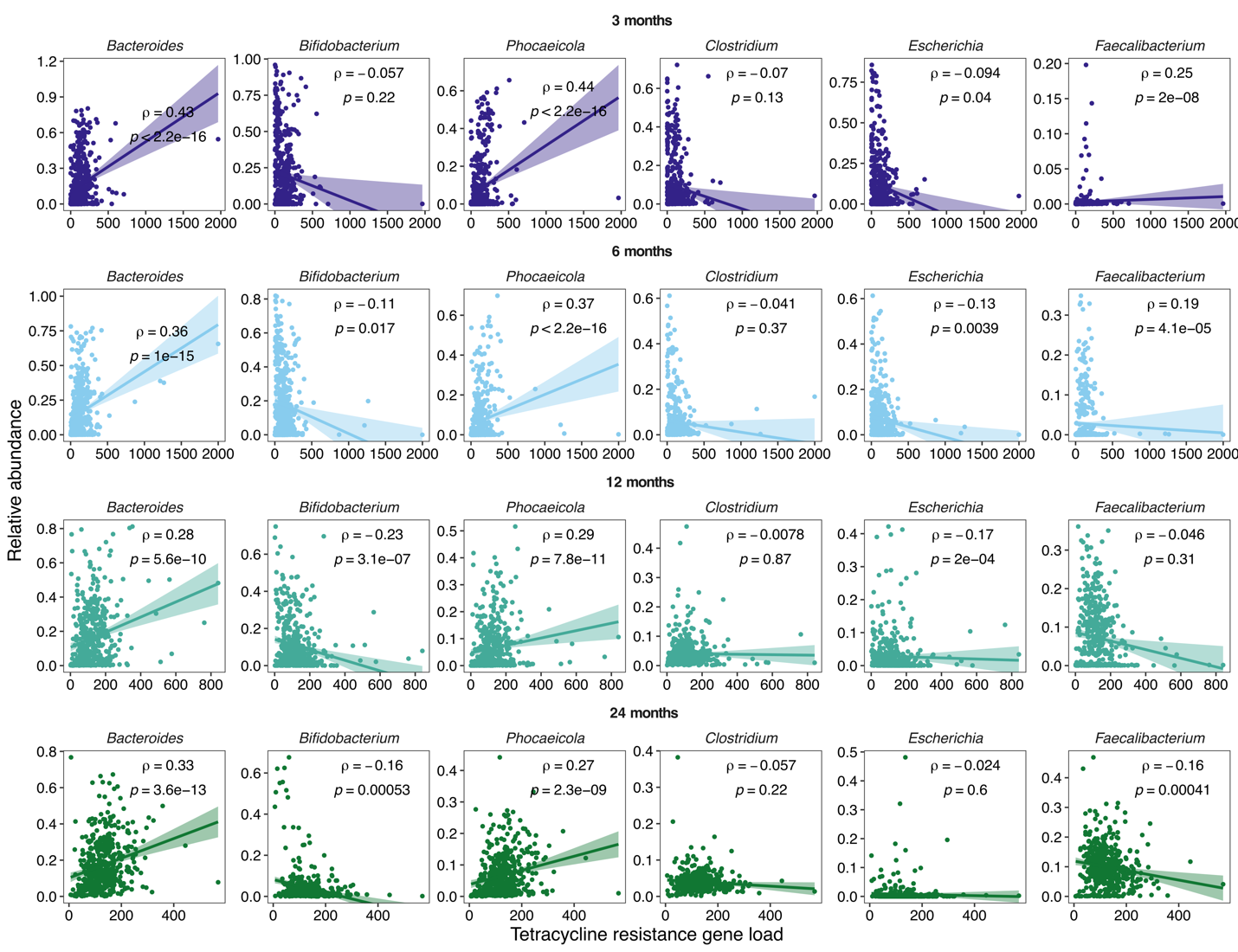


**Supplementary Figure S7 Correlations between tetracycline resistance gene load and 6 most abundant genera**. Correlations calculated with Sperman’s rank correlation.


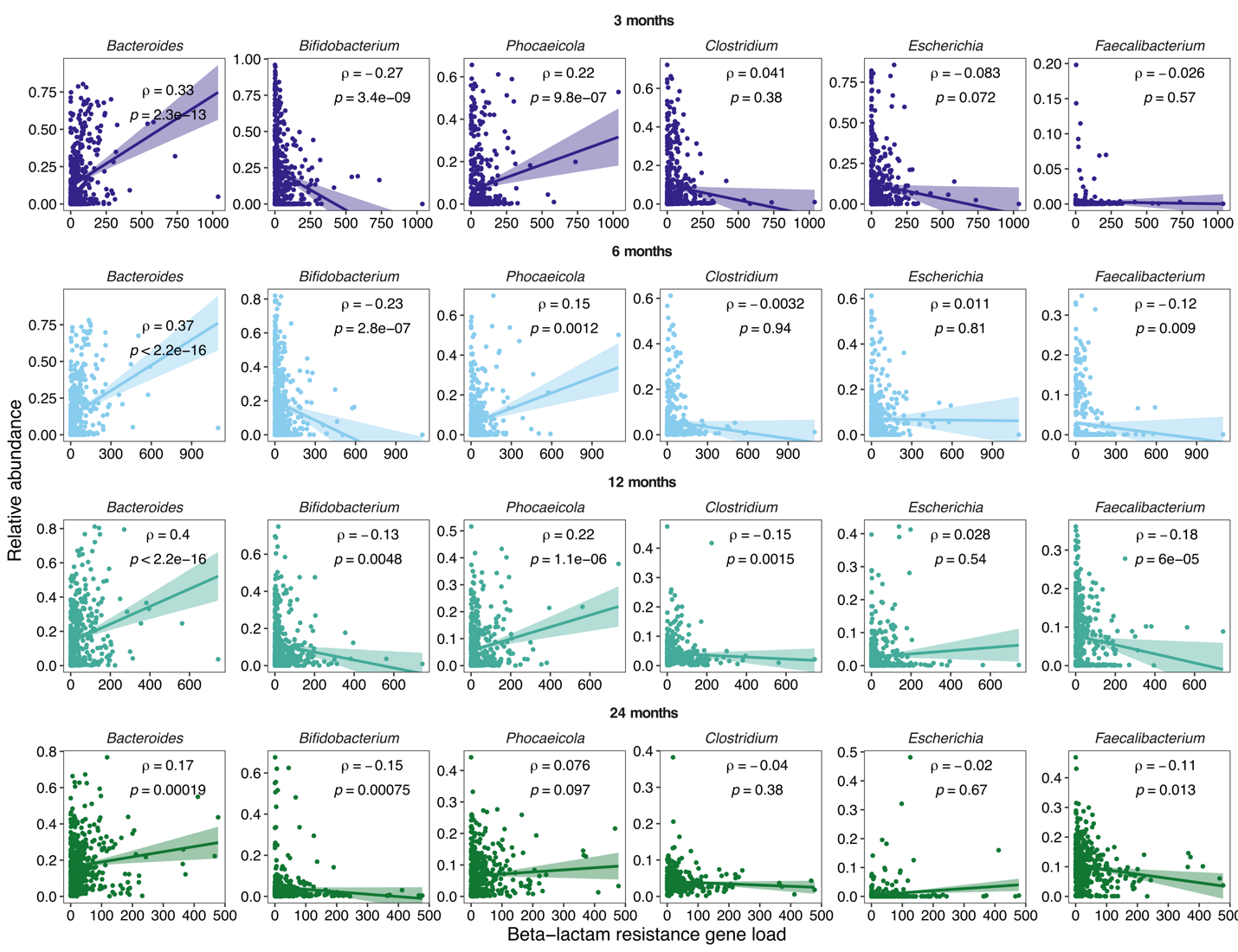


**Supplementary Figure S8 Correlations between beta-lactam resistance gene load and 6 most abundant genera.** Correlations calculated with Sperman’s rank correlation.

**
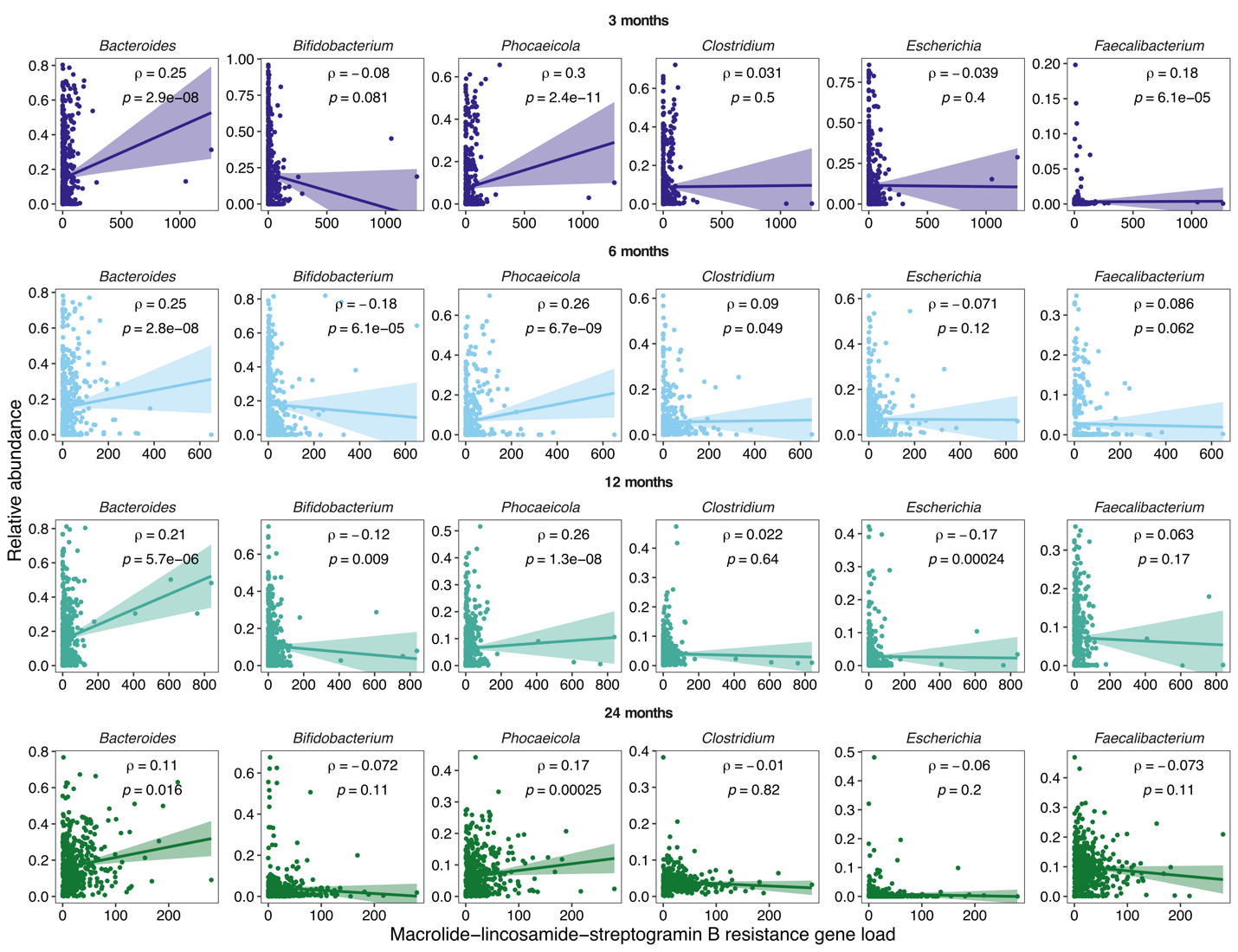
**

**Supplementary Figure S9 Correlations between macrolide-lincosamide-streptogramin B resistance gene load and 6 most abundant genera.** Correlations calculated with Sperman’s rank correlation.


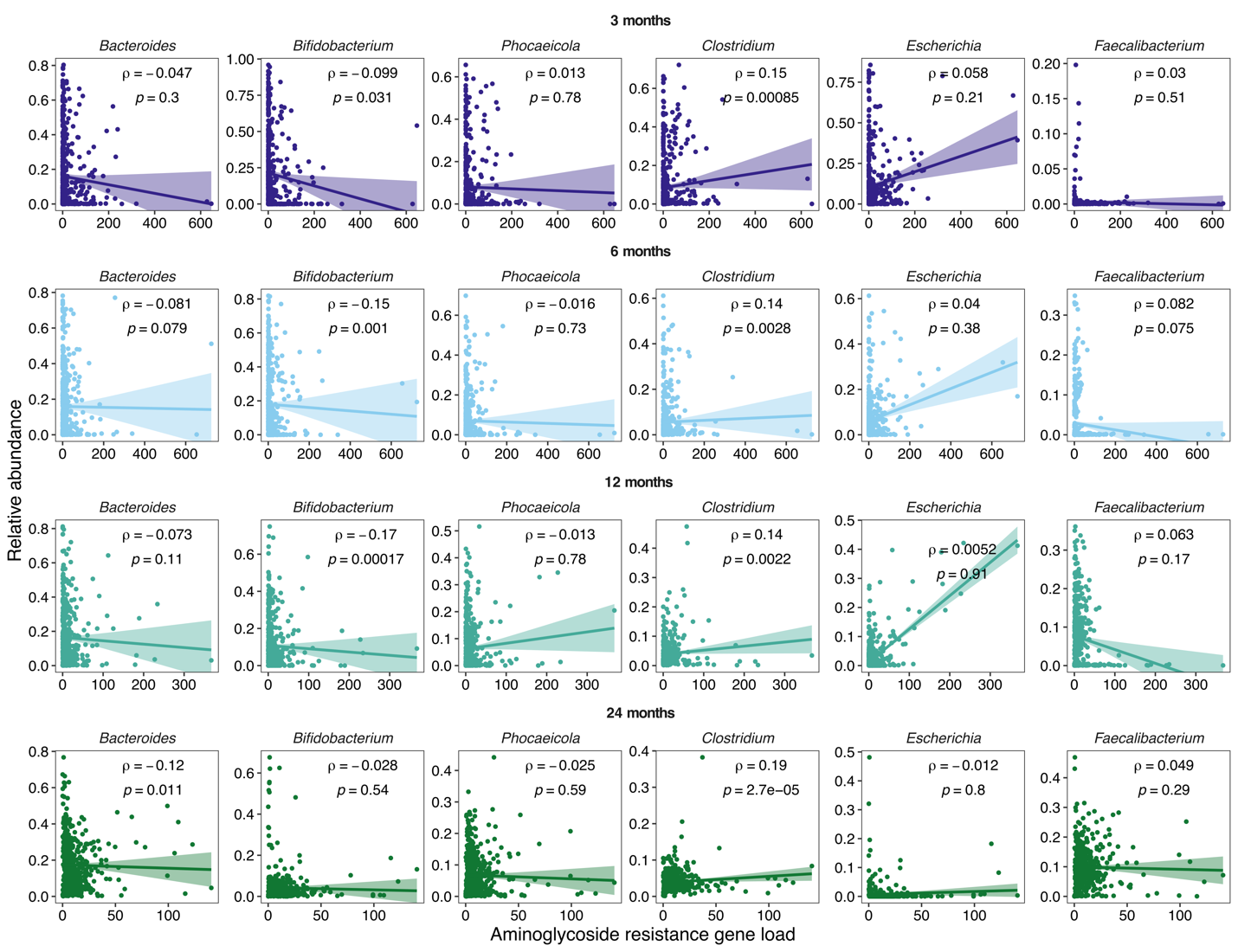


**Supplementary Figure S10 Correlations between aminoglycoside resistance gene load and 6 most abundant genera.** Correlations calculated with Sperman’s rank correlation.


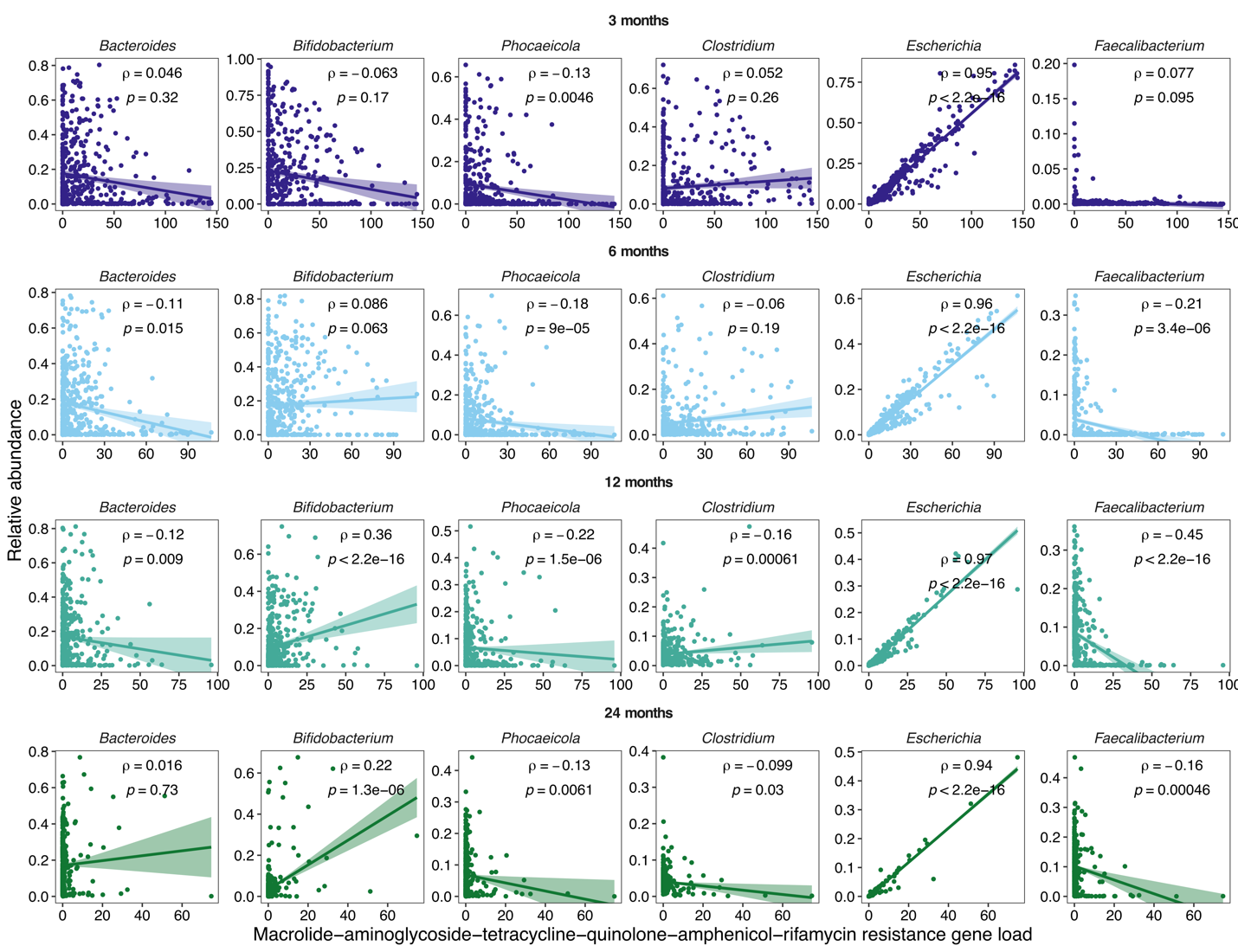


**Supplementary Figure S11 Correlations between macrolide-aminoglycoside-tetracycline-quinolone-amphenicol-rifamycin resistance gene load and 6 most abundant genera.** Correlations calculated with Sperman’s rank correlation.

## Supplementary Tables:

Supplementary Table 1 Differences of delivery related variables and bacterial community types.

|  | Community type |  | Delivery mode | | Intrapartum antibiotics | | Parity |  |
| --- | --- | --- | --- | --- | --- | --- | --- | --- |
| Months |  | All | Vaginal | Caesarean | No | Yes | Nulli- | Multi- |
| **3** | Bif+Bac | 208 (44%) | ***198 (50%)*** | ***10 (12%)*** | ***162 (54%)*** | ***46 (26%)*** | ***79 (34%)*** | ***129 (52%)*** |
|  | Clo+Ent | 185 (39%) | ***135 (34%)*** | ***50 (61%)*** | ***92 (31%)*** | ***93 (53%)*** | ***109 (48%)*** | ***76 (31%)*** |
|  | Bif+Ent | 82 (17%) | ***61 (15%)*** | ***21 (26%)*** | ***44 (15%)*** | ***38 (21%)*** | ***41 (18%)*** | ***41 (17%)*** |
| **6** | Bif+Bac | 282 (59%) | ***250 (63%)*** | ***32 (40%)*** | *195 (65%)* | *87 (49%)* | 125 (55%) | 157 (64%) |
|  | Clo+Ent | 97 (20%) | ***74 (19%)*** | ***23 (28%)*** | *56 (19%)* | *41 (23%)* | 54 (24%) | 43 (17%) |
|  | Bif+Ent | 96 (20%) | ***70 (18%)*** | ***26 (32%)*** | *47 (16%)* | *49 (28%)* | 50 (22%) | 46 (19%) |
| **12** | Bif+Ent+Vei | 297 (63%) | 250 (63%) | 47 (58%) | 184 (62%) | 113 (64%) | 148 (65%) | 149 (61%) |
|  | Lac+Osc | 178 (37%) | 144 (37%) | 34 (42%) | 114 (38%) | 64 (36%) | 81 (35%) | 97 (39%) |
| **24** | Bif+Ent+Vei | 97 (20%) | 84 (21%) | 13 (16%) | 66 (22%) | 31 (18%) | *59 (26%)* | *38 (15%)* |
|  | Lac+Osc | 378 (80%) | 310 (79%) | 68 (84%) | 232 (78%) | 146 (82%) | *170 (74%)* | *208 (85%)* |

Due to rounding, some of the percentages do to sum up to 100%. Italicised results mark P-value of <0.01 and italicised and bolded results a P-values of <0.001 calculated with c^2^ test.
